# The Unique Non-Catalytic C-Terminus of P37delta-PI3K Adds Proliferative Properties *In Vitro* and *In Vivo*

**DOI:** 10.1371/journal.pone.0127497

**Published:** 2015-05-29

**Authors:** Katarina Ejeskär, Oscar Vickes, Arunakar Kuchipudi, Yvonne Wettergren, Anne Uv, Victoria Rotter Sopasakis

**Affiliations:** 1 Systems Biology Research Center, School of Bioscience, University of Skövde, Skövde, Sweden; 2 Department of Medical and Clinical Genetics, Gothenburg University, Gothenburg, Sweden; 3 Department of General Surgery, Gothenburg University, Gothenburg, Sweden; 4 Department of Molecular and Clinical Medicine, Institute of Medicine, Wallenberg Laboratory, Gothenburg University, Gothenburg, Sweden; Florida International University, USA

## Abstract

The PI3K/Akt pathway is central for numerous cellular functions and is frequently deregulated in human cancers. The catalytic subunits of PI3K, p110, are thought to have a potential oncogenic function, and the regulatory subunit p85 exerts tumor suppressor properties. The fruit fly, *Drosophila melanogaster*, is a highly suitable system to investigate PI3K signaling, expressing one catalytic, Dp110, and one regulatory subunit, Dp60, and both show strong homology with the human PI3K proteins p110 and p85. We recently showed that p37δ, an alternatively spliced product of human PI3K p110δ, displayed strong proliferation-promoting properties despite lacking the catalytic domain completely. Here we functionally evaluate the different domains of human p37δ in *Drosophila*. The N-terminal region of Dp110 alone promotes cell proliferation, and we show that the unique C-terminal region of human p37δ further enhances these proliferative properties, both when expressed in *Drosophila*, and in human HEK-293 cells. Surprisingly, although the N-terminal region of Dp110 and the C-terminal region of p37δ both display proliferative effects, over-expression of full length Dp110 or the N-terminal part of Dp110 decreases survival in *Drosophila*, whereas the unique C-terminal region of p37δ prevents this effect. Furthermore, we found that the N-terminal region of the catalytic subunit of PI3K p110, including only the Dp60 (p85)-binding domain and a minor part of the Ras binding domain, rescues phenotypes with severely impaired development caused by Dp60 over-expression in *Drosophila*, possibly by regulating the levels of Dp60, and also by increasing the levels of phosphorylated Akt. Our results indicate a novel kinase-independent function of the PI3K catalytic subunit.

## Introduction

Phosphoinositide 3-kinases (PI3K) are a family of lipid kinases that activate a signaling cascade with subsequent activation of the Akt kinase, which mediates various responses such as growth, metabolism, survival and migration[1]. Human Type I_A_ PI3K is a heterodimer composed of a p85 regulatory subunit in complex with a p110 catalytic subunit. Different p110 isoforms appear to be differentially expressed and to exhibit distinct functions[2]. Whereas p110α and p110β show a ubiquitous expression pattern, p110δ is mainly expressed in leukocytes[3, 4], and in the embryonic nervous system[5]. The p110α subunit is frequently mutated in tumors[6], the p110β and p110δ isoforms on the other hand are often over-expressed in tumors[7, 8]. Recent studies have shown that p85α can act as a tumor suppressor[9], and that low expression of p85α in breast cancer lead to poor survival[10], while some mutations makes p85 act as an oncogene[11]. The tumor suppressor lipid phosphatase Pten can down-regulate the PI3K pathway, and Pten is commonly altered in tumors[12]. The PI3K regulatory subunit p85α can bind to Pten and enhance its stability/activity[13–15], making the monomeric form of p85α an important negative regulator of the PI3K signaling pathway. Moreover, it has been shown that over-expression of the PI3K catalytic isoform p110δ dampens Pten activity[16].

PI3K in *Drosophila melanogaster* is composed of a catalytic subunit, called Dp110, and a regulatory subunit that corresponds to human p85, called Dp60. Both proteins show high homology to the corresponding human proteins[17]. Previous studies have shown that altered expression of Dp110 or Dp60 in *Drosophila* influences larval growth and size of the imaginal discs[17, 18], with elevated PI3K signaling resulting in vastly increased growth of the organism[19]. The PI3K pathway has also been implicated in the life span of *Drosophila*. Life expectancy is extended by more than 50% when the insulin-like receptor (InR) [20] or its receptor substrate (chico/IRS) [21] are mutated, and this was mediated by dFOXO[22]. In addition, life span increases when PI3K is pharmacologically suppressed[23]. PI3K signaling is also involved in programmed- and starvation-induced fat body autophagy in *Drosophila*, repressing hormone-induced autophagy[24].

We recently identified p37δ, an alternatively spliced product of human *PIK3CD* that encodes p110δ. The p37δ isoform encompasses the N-terminal p85-binding domain and a small part of the RAS-binding domain of p110δ, as well as a unique 100 amino acid C-terminal part. p37δ has proliferation-promoting properties, despite lacking a catalytic domain, and possibly participates in PI3K/Akt signaling through interactions with the PI3K regulatory subunit p85 or in RAS-signaling by stabilizing RAS proteins[25]. Elevated levels of p37δ-mRNA and protein were detected in ovarian, colorectal and neuroblastoma tumors[25, 26]. Here, we evaluate the function of different domains of human p37δ using human cell cultures and *Drosophila*. We conclude that the N-terminal region of p110/p37δ, including the p85-binding domain and part of the RAS-binding domain, can partly rescue Dp60 over-expression phenotypes and induces phosphorylation of Akt, whereas the unique C-terminal part of p37δ is necessary for promoting increased proliferation, suggesting that p37δ triggers PI3K signaling despite lacking a catalytic domain.

## Results

### The unique C-terminal domain of human p37δ is crucial for its ability to induce proliferation of HEK-293 cells

**Fig 1:**
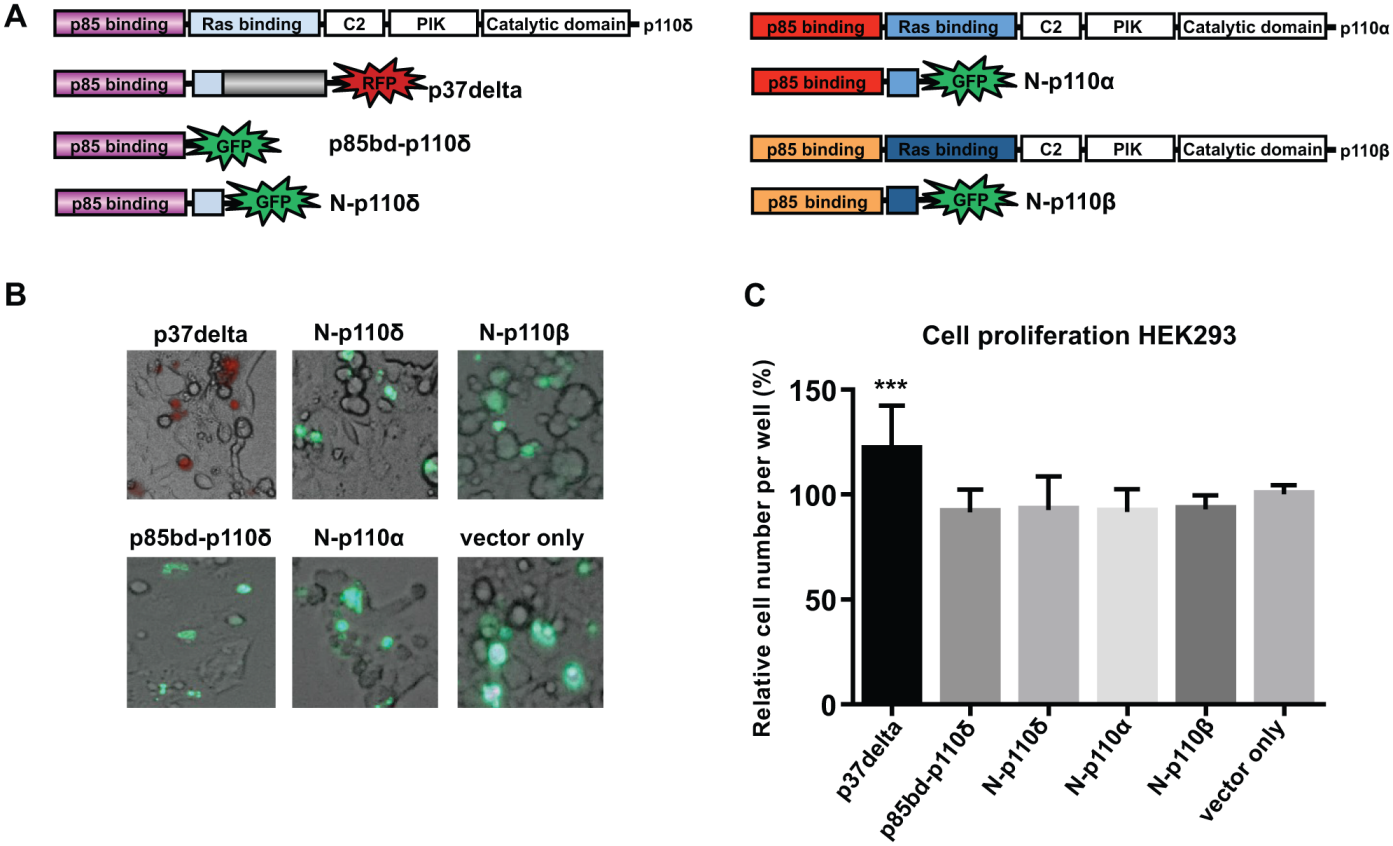
The C-terminal part of p37δ is needed for increased proliferation *in vitro*. (A) Schematic drawing of constructs used, cloned into the pAcGFP-N1 vector. (B) Fluorescent microscopy photos showing the transfection efficiency of the different constructs. (C) Graph showing relative number of cells compared to control 48 hours after transfection, average of three different experiments. There are significantly more cells in the p37delta experiment compared to all other constructs.

We have previously shown that expression of p37δ in HEK-293 cells causes increased cell proliferation[25]. To address if the unique C-terminal part of p37δ might contribute to this effect, we compared the proliferation rate of HEK-293 cells expressing p37δ to those expressing only the N-terminus of p37δ that is identical to the N-terminal region of the full-length p110δ. Thus, green (GFP) or red (RFP) fluorescent protein fusion constructs of p37δ and of the N-terminal part of human p110δ/p37δ (N-p110δ) were expressed in HEK-293 cells by transient transfection (Fig 1A). Transfection efficiency was similar between experiments, as shown by fluorescent microscopy (Fig 1B), and cell proliferation experiments were compared to control (vector only) by calculating the number of living cells 48 hours after transfection (Fig 1C). The p37δ expressing cells grew significantly faster than those expressing N-p110δ (P = 0.0002), and the proliferation rate of cells expressing N-p110δ was similar to control cells. In line with these results, we found that expression of the N-terminal part of p110α or p110β (corresponding to the p110δ-part of p37δ) or expression of the p85-binding domain of p110δ alone resulted in no increase in cell proliferation compared to control cells (Fig 1). This suggests that the unique C-terminal part of p37δ is crucial for its proliferative properties in HEK-293 cells.

### N-p110 promotes cell proliferation in Drosophila, and the unique C-terminal domain of human p37δ enhances the effect

**Fig 2:**
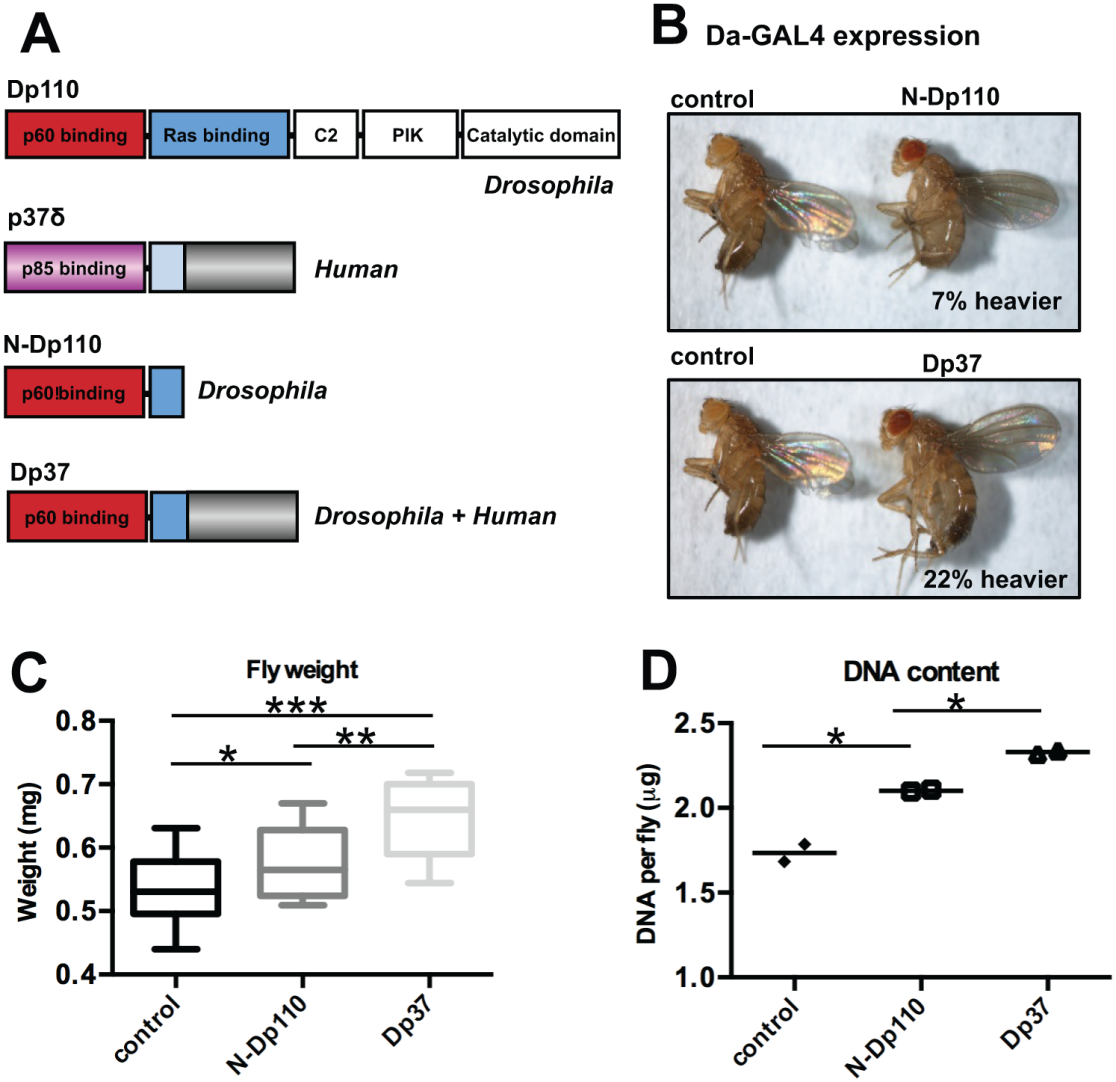
The C-terminal part of human p37δ adds proliferative effect in *Drosophila*. (A) Schematic drawing of constructs cloned into the pUASattB vector. (B) Photo of two-day-old male control flies (left) compared to N-Dp110 (top) or Dp37 (bottom) expressing flies. (C) Average weight (mg) per two day old fly, n = 100. Significance levels indicated at the top. (D) Average DNA content (μg) per fly. Four flies measured each time in two different experiments. Significance indicated at the top.

Given the differential effects of p37δ and N-p110δ on cell proliferation *in vitro*, we addressed their effects *in vivo*, using *Drosophila melanogaster*. Flies were generated that expressed either the N-terminal part of *Drosophila* p110 (N-Dp110), corresponding to human N-p110δ, or the N-Dp110 combined with the C-terminal part of human p37δ (Dp37) under control of the UAS-promoter (Fig 2A). Homozygous UAS-flies were crossed to Daughterless-GAL4 (Da-Gal4) flies resulting in offspring ubiquitously expressing N-Dp110 or Dp37. Overexpression of N-Dp110 resulted in 7% (P = 0.02) increased weight of the male flies, while expression of Dp37 resulted in 22% (p<0.0001) increased weight (Fig 2B and 2C). Flies carrying one copy of Da-GAL4 served as control. The larger weight of the flies corresponded to an increase in total cell number, since the average DNA content of the flies expressing N-Dp110 (2.1 μg DNA/fly) or Dp37 (2.3 μg DNA/fly) was higher than in control flies (1.7 μg DNA/fly) (P = 0.01) (Fig 2D). The flies looked otherwise phenotypically normal (Fig 2B). Interestingly, the Dp37-expressing flies were heavier (P<0.001) and had a higher DNA-content (P = 0.003) than N-Dp110-expressing flies (Fig 2C and 2D), suggesting that the unique C-terminal part of human p37δ contributes significantly to its cell-proliferative effect *in vivo*.

### Over-expression of full length Dp110 or N-Dp110, but not p37δ, decreases life span

**Fig 3:**
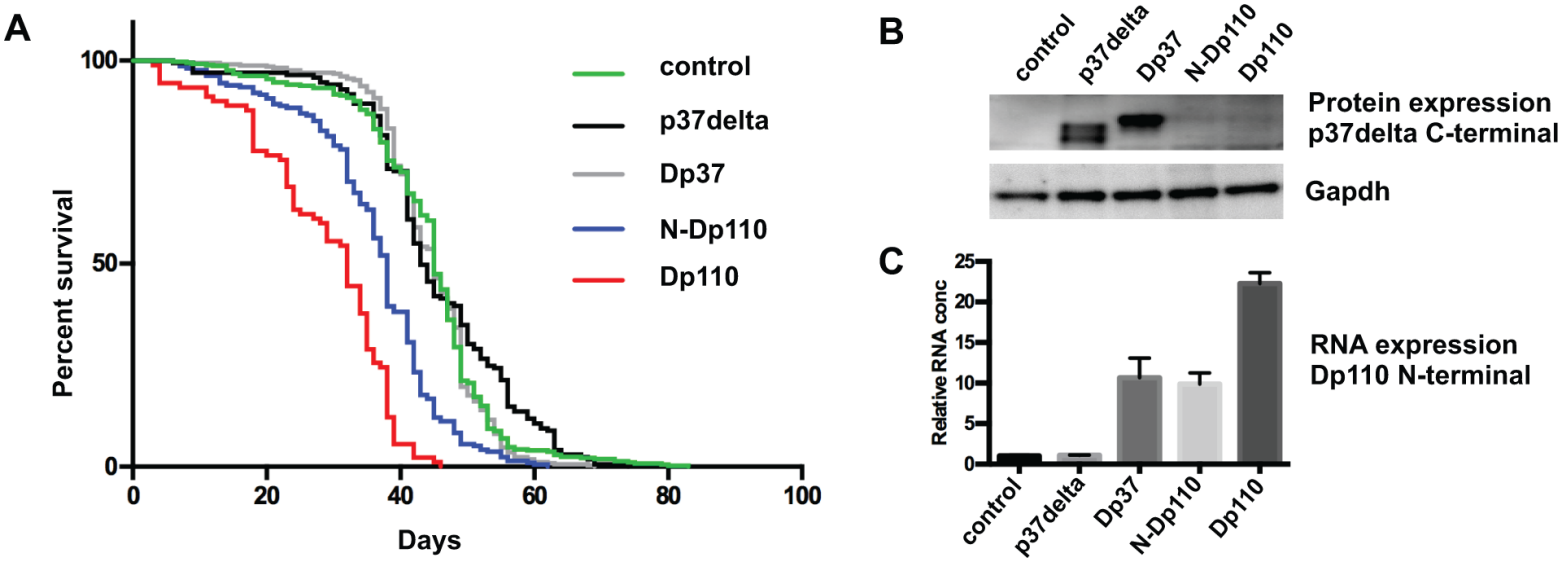
Expression of N-terminal part of Dp110 shortens the life span. (A) Kaplan Meier curve, showing the life span of flies at 25°C over-expressing constructs indicated at the right. (B) Western blots of *Drosophila* proteins, Da-Gal4 expression. Antibody indicated to the right. (C) Quantitative PCR of N-terminal Dp110, Pi3k92E RNA. Average RNA expression level compared to control.

Dp110 participates in insulin signaling, thereby affecting metabolism, growth and longevity[27]. In longevity studies of male flies, we found that expression of full length Dp110, using the Da-GAL4 driver line, decreased median life span by 29% (from 45 days to 32 days, P < 0.0001) (Fig 3A). To test if p37δ expression also can cause changes in life span, we similarly over-expressed human p37δ, N-Dp110 or Dp37. The median survival of p37δ- (43 days) and Dp37- (45 days) expressing flies was similar to control flies (45 days). However, the median survival of N-Dp110 (38 days) was significantly lower than that of the control (P < 0.0001) (Fig 3A), approaching the effect of full length Dp110. The equal expression level for those constructs that includes the C-terminal part of p37δ (p37δ and Dp37) was verified by Western blot using a p37δ C-terminal specific antibody (Fig 3B). The constructs including the N-terminal part of *Drosophila* Dp110 (Dp37, N-Dp110 and Dp110) the level of over expression was verified by qPCR. Here equal overexpression levels were confirmed for Dp37 and N-Dp110, the full length Dp110 over-expression flies showed higher expression (Fig 3C).

Thus, the increase in growth caused by expression of Dp37 does not appear associated with a decrease in life span, as observed for N-Dp110.

### Expression of the non-catalytic Dp37 and p37δ in vivo results in marked Akt phosphorylation and rescues the embryonic lethality of Dp60 over-expression

**Fig 4:**
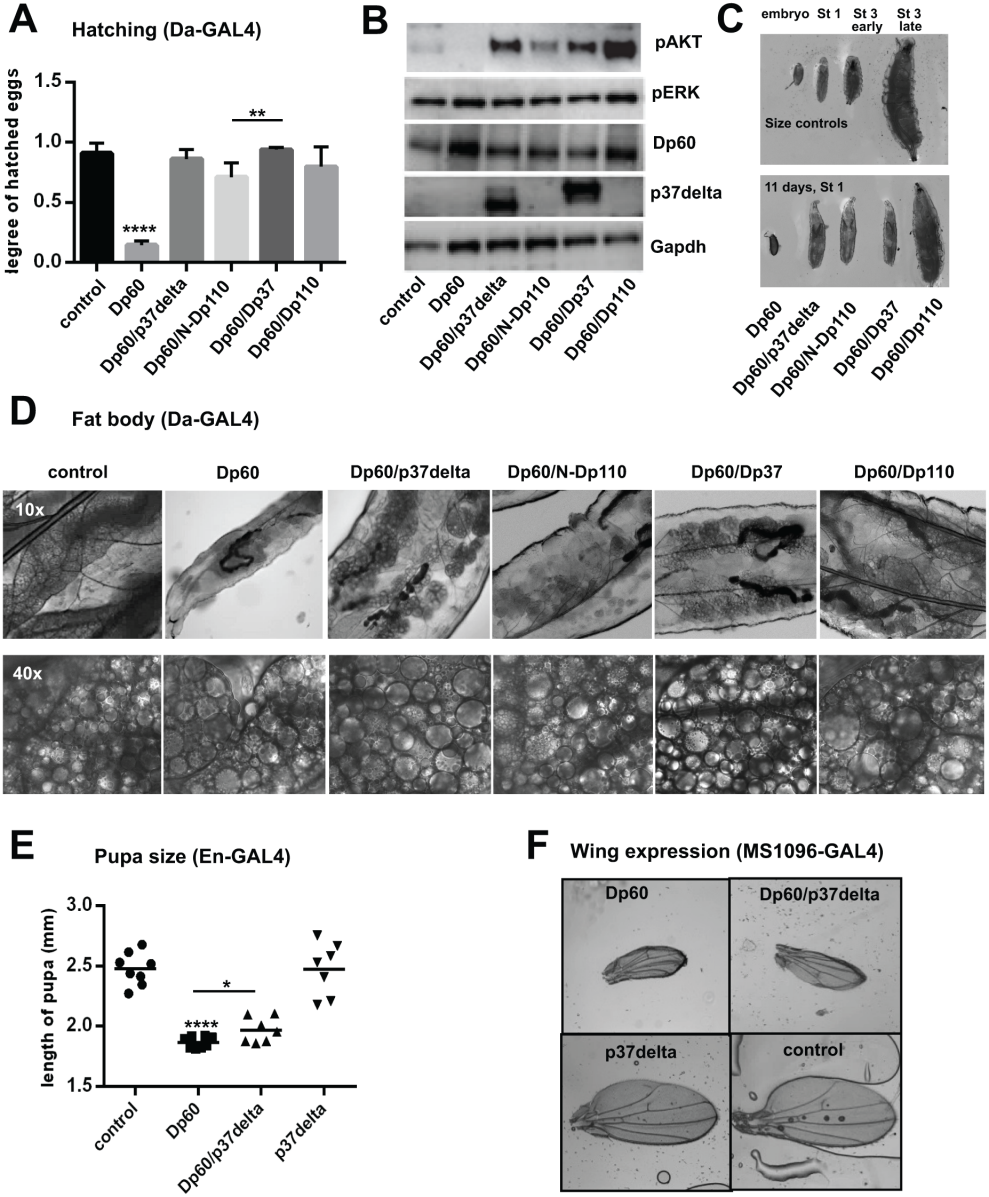
The N-terminal part of Dp110 rescues Dp60 over expression phenotypes. (A) Graph showing the degree of embryos with expression driven by Da-Gal4, which hatched into larvae. Dp60 expressing embryos significantly differ from all others. (B) Western blots of *Drosophila* embryo proteins, Da-Gal4 expression. (C) Photo of larvae. Size controls (Da-Gal4) of different stages and embryo, at the top. Dp60 co-expressing larvae 11 days after hatching at the bottom to the right, Dp60 expressing 11 day old embryo to the left. (D) 10x (top) and 40x (bottom) magnification of fat body in larvae, gene expression by Da-Gal4, showing severely disorganized fat body. For the control and Dp60 expressing larvae photo was taken 4 days after hatching. All Dp60 co-expressing larvae are 1^st^ instar, photo taken 11 days after hatching. (E) Graph showing pupa length, En-Gal4 expression. (F) Photos of wings from two-day-old flies, MS1096-Gal4 expression in the wing-disc.

To further understand the differential effects of N-Dp110 and Dp37 on proliferation and life span, we asked if their functional effects are coupled with the downstream PI3K signaling pathway. The p110 subunit of PI3K is regulated by the adaptor protein p85, called Dp60 in *Drosophila*[28]. While over-expression of Dp110 causes increased growth[17], over-expression of Dp60 appears to arrest growth at the 1^st^ instar larvae[29]. We tested if simultaneous expression of N-Dp110 or Dp37 with Dp60 had any effect on the Dp60 over-expressing phenotype. When Da-Gal4 was used to drive ubiquitous expression of Dp60, we found that the majority of the embryos failed to hatch. Only 15% ± 2% of embryos developed into crawling first instar larvae, compared to 91% ± 4% for control embryos (Fig 4A). These embryos showed elevated levels of Dp60 as expected, and undetectable levels of phosphorylated Akt (pAkt), a downstream target of PI3K signaling (Fig 4B). Co-expression of either p37δ, N-Dp110, Dp37 or Dp110 rescued embryonic lethality. The strongest rescuing ability was observed for Dp37 (94%±1%) and p37δ (86%±4%), but co-expression with N-Dp110 (71%±6%) or Dp110 (80%±7%) also significantly increased the rate of hatching embryos (P<0.0001) (Fig 4A). Furthermore, all four p110 variants, including the non-catalytic Dp37 and p37δ, also resulted in increased levels of pAkt (Fig 4B), compared to control animals, the increased Akt phosphorylation was accompanied by reduced levels of Dp60 compared to embryos that only express Dp60 (Fig 4B). Thus, both N-Dp110 and Dp37 can stimulate phosphorylation of Akt and repress embryonic lethality induced by Dp60 expression, despite lacking a catalytic domain. Previous studies showed that over-expression of p37δ alone affects the phosphorylation level of Erk[25], a downstream target of Ras, however the levels of phosphorylated Erk was unaffected in all embryos with Dp60 over-expression (Fig 4B).

### Expression of the N-terminal region of p110 is sufficient to rescue larvae growth inhibition caused by over-expression of the adaptor subunit Dp60, but not larvae development or fat body abnormalities

The few Dp60-expressing larvae that hatched lived for 5–7 days as 1^st^ instar larvae without growing in size. These small larvae also exhibited morphological anomalies in the fat body with larger lipid droplets forming in the adipocytes (Fig 4D). When Dp60 was co-expressed with either of the Dp110-related variants, the hatching larvae remained as 1^st^ instar for around 2 weeks until they died and exhibited a similar abnormal morphology of the fat body. However, co-expression with the p110 variants enabled the larvae to grow in size, reaching that of a young 3^rd^ instar larvae (Fig 4C). Thus, expression of Dp110, Dp37, N-Dp110 and p37δ were not able to rescue the moulting defect or abnormal fat body morphology caused by over-expression of Dp60, but had a dramatic effect on the growth inhibition caused by Dp60 expression (Fig 4C and 4D). When Dp60 was co-expressed with the full length Dp110, the larvae grew to the size of a late 3^rd^ instar (Fig 4C). Some of these larvae also developed into pupae, however no flies eclosed.

### p37δ partly rescues the Dp60 engrailed-controlled pupae size phenotype, but not the MS1096-controlled Dp60 wing phenotype

When over-expressing Dp60 in segments of the fly using the engrailed (En)-Gal4 promoter, the larvae were able to develop to the pupae stage, but no flies eclosed from either Dp60 over-expression pupae or pupae co-expressing Dp60 with p37δ. The Dp60 expressing pupae were very small (1.87 mm) compared to control (2.48 mm) or p37δ-expressing (2.48 mm) pupae (P<0.0001). Co-expressing Dp60 with p37δ resulted in an increase in pupa size (1.97 mm) compared to Dp60 alone (P = 0.02) (Fig 4E).

Expressing Dp60 or co-expressing Dp60 with p37δ in the wing disc using the MS1096-Gal4 driver, resulted in smaller wings with curly edges (Fig 4F), and co-expression with p37δ did not rescue the phenotype. Over-expression of p37δ alone did not affect the size or shape of the wing (Fig 4E), suggesting that kinase activation of the catalytic subunit is necessary for wing development in *Drosophila*.

Taken together, the N-terminal part of p110 has unique and specific proliferative properties that are separate from the catalytic function of this protein that are powerful enough to rescue severe phenotypic characteristics of Dp60 over-expressing flies, possibly by lowering the levels of Dp60 and increasing the levels of pAkt.

## Discussion

We have previously shown that over-expression of p37δ promotes cell proliferation in *Drosophila*[25]. Here we investigated the functional role of the N-terminal part of *Drosophila* p110 (Dp110), corresponding to the N-terminal part of human p37δ as well as the role of the unique C-terminal part of human p37δ, not detected in other proteins. The N-terminal part of Dp110 had a similar growth-promoting effect in *Drosophila* as p37δ (7% heavier flies) (Fig 2), although addition of the C-terminal part of human p37δ to the fly-specific Dp110, resulted in a much larger increase in growth (22% heavier flies) (Fig 2). Thus the C-terminal part seems to have an important and unique cell-proliferative function, albeit we cannot find any similar domains in other proteins by similarity searches (BLAST). This part of the protein includes a long sequence of intron five, which explains why it is not conserved in other related proteins. The potentiating effects of the unique C-terminal part of p37δ in combination with the *Drosophila* p110 N-terminal domain compared to using the C-terminal part in combination with the human N-terminal domain might simply be due to better interaction abilities with other *Drosophila* proteins when using the *Drosophila* Dp110. We hypothesized that expressing the N-terminal part of human p110α, p110β or p110δ would have a proliferative effect *in vitro*, since we observed an effect on the N-terminal part of *Drosophila* p110 (N-Dp110) on proliferation (Fig 2). However, we found no effects on proliferation of the N-terminus of these mammalian isoforms in human HEK-293 cells (Fig 1). It’s possible that the *Drosophila* p110 N-terminal part involves additional functions, despite high homology to the human isoforms. Mammalian PI3Ks constitute a large family of proteins divided into three main classes, allowing more specificity for each family member, whereas the fruit fly only has one PI3K that would logically have to involve a broader spectrum of functions in growth and metabolism.

It is intriguing that the decreased survival rate is close to identical between flies expressing N-Dp110 and flies expressing full length Dp110 (Fig 3A), the small difference in survival between the flies overexpressing these constructs could be due to the expression of the full length Dp110 being higher compared to the expression of N-Dp110 (Fig 3C). The fact that overexpression of N-Dp110 decreased the survival suggests that the increased survival seen when PI3K is suppressed[23] might be caused by its non-catalytic properties. Interestingly, the effect on survival was lost when the C-terminal part of p37δ was added to the protein, indicating that the proliferative properties and the ability of PI3K to affect life span are two separate functions of the PI3K catalytic subunit p110.

By co-expressing N-terminal constructs of Dp110, or full length Dp110 with Dp60 in the fly embryo, the severe developmental defects caused by Dp60 alone could partly be rescued (Fig 4), probably due to the Dp60 (p85)–binding properties of all constructs. Still, severe defects remained, making a development halt at 1^st^ instar larval stage, and a highly disorganized fat body (Fig 4D). Another interesting finding was that co-expression of N-terminal constructs of Dp110 together with Dp60, resulted in dramatically increased levels of phosphorylated Akt, similar to levels obtained with full length Dp110 (Fig 4B), indicating that the p110 proteins can trigger PI3K pathway response independently of its catalytic function. The mechanism behind this kinase-independent phenomenon could be due to the ability to bind to the regulatory subunit p85 (Dp60 in the fly) and regulate the levels and/or cellular localization of p85[25]. This might in turn affect the activity of Pten negatively, as p85 has been shown to enhance Pten activity[13–15].

In summary, we show that the unique C-terminal part of human p37δ enhances the proliferative properties of the p37δ protein, both when expressed in *Drosophila*, and in human HEK-293 cells. We found that the N-terminal part of the catalytic subunit of PI3K p110, including only the Dp60 (p85)-binding domain and a minor part of the Ras binding domain, rescues severe phenotypes of Dp60 over-expression, possibly by regulating the levels of Dp60, and also by increasing the levels of phosphorylated Akt. This indicates possibilities for all p110 variants to regulate the PI3K pathway in other ways than through PIP2 phosphorylation.

## Materials and Methods

### Plasmids

N-terminal parts of *PIK3CD_v2* (p37δ-mRNA) (GenBank: JN190435), *PIK3CA* (NM_006218.2) and *PIK3CB* (NM_006219) were amplified from human cDNA and cloned into pDsRed-Monomer-N1 and pAc-GFP1-N2 vectors (Clontech), creating constructs with dsRed/GFP1-fusion tags. *PIK3CD_v2* ^*p*37δ^ and *PiK92E* sequences (S1 Fig) were cloned into the pUASTattB vector[30].

All constructs were verified by BigDye Cycle sequencing (Applied Biosystems).

### Transfection and cell proliferation

HEK-293 cells were grown in DMEM with 10% FBS and transfected according to standard protocol using Lipofectamine2000 (Invitrogen). Transfection efficiency was estimated using fluorescence microscopy after each experiment. For proliferation experiments cells were seeded in quadruplicates in 96-well plates. Viability was monitored during four days post transfection using CellTiter96 Aqueous One Solution (Promega). All experiments were repeated three times.

### Fly strains

The *pUASTattB* constructs encoding p37δ, N-Dp110 or Dp37 (Fig 2A) were inserted at a predetermined site in the fly genome, using the phiC31-based integration system and *PBac{y* ^*+*^ *-attP-3B}VK00033* as docking position, by BestGene Inc. Control flies carrying the empty docking site, *UAS-Dp110* and *UAS-Dp60* and the *da-GAL4*, *en-Gal4* and *MS1096-Gal4* driver flies were obtained from Bloomington Stock Center.

### Fly weight and DNA content

For weight measurements, 20 female *da-GAL4* and 9 male UAS-transgenic flies (or control flies) were mated. Offspring were collected for 24 hours at 25°C and then reared at 29°C until eclosion. Male F1 progeny were collected every 24 hours and aged for 2 days before measurements. Three parallel crosses were set up for each genotype, and offspring were collected from each cross for four consecutive days. Pools of 5 flies were weighed on a precision balance and the average weight per fly was calculated (n = 100 for each cross). The experiment was repeated twice on different occasions. To calculate total DNA mass per fly, 10 pools of 5 males from each cross were weighed, and their DNA was extracted using QIAamp DNA Micro Kit (Qiagen). DNA concentration was measured using spectrometry.

### Survival studies

*Da-Gal4* females were crossed to males with UAS-constructs according to (Fig 3). For each cross 150–200 males were collected and kept, 20 in each, in standard vials at 25°C, the number of dead flies were counted every second day, all vials were shifted twice a week. The experiment was repeated at two separate occasions.

### Hatching studies

*Da-Gal4* females were crossed to control (white) males or males with UAS-Dp60-constructs according to (Fig 4A), and caged on apple plates for 6 hours at 25°C. The number of eggs was counted and the plates were then kept at 29°C for 48 hours, following counting of number of not hatched embryos. The experiment was repeated at four different occasions, and the average degree of hatching was calculated.

### Protein isolation, SDS-PAGE, western blot and antibodies

Embryos (stage 13 to 17), or adult flies were homogenized in Western blot loading buffer supplemented with HALT Phosphatase and Protease inhibitor cocktail (Pierce, Rockford, IL). Western blot were performed according standard procedures using rabbit polyclonal antibodies to gapdh (sc-25778) from Santa Cruz Biotechnology (Dallas, TX), Drosophila pAKT (4054) and pErk1/2 (9101) from Cell Signaling Technology (Danvers, MA), and p85α (06–496) from Millipore, while C-terminal p37δ—specific polyclonal antibodies were produced in house from rabbit (Agrisera, Vännäs, Sweden). Performance and specificity of this antibody has been described previously [25].

### RNA isolation and qPCR

Total RNA was extracted from adult flies using RNeasy Mini kit (Qiagen). cDNA was synthesized from 1μg of total RNA using High capacity RNA to cDNA kit (Applied Biosystems). We used Perfecta SYBR green FastMix (Quanta Biosciences), with Pik392E fp: GTGTACGGCATCTCGACCTT, and Pi3k92E rp: AGTCGCTGCTAAAGCTCGTT in the real time qPCR reaction. All according to suppliers protocol. The qPCR was performed and analyzed using the Thermo Scientific PikoReal Real-time PCR System (Fisher Scientific). All RNA expression experiments were in triplicates and compared to control expression (white x Da-Gal4-flies).

### Fat body, pupa size and wing expression

For fat body studies, da-Gal4 females were crossed to control males or male with UAS-constructs according to (Fig 4D). The larvae were kept at 25°C, in standard vials for 4–12 days (for control and Dp60 larvae, 4 days). The fat body and larval stage were documented in a microscope using 10x and 40x magnification. En-Gal4 females were crossed to control males or male with UAS-constructs according to (Fig 4E). The flies were kept at 25°C and the length of the pupae were measured. For expression in the *Drosophila* wing, MS1096-Gal4 females were crossed to control males or males with UAS-constructs according to (Fig 4F). The flies were kept at 25°C, and the wings were documented two days after eclosure. All experiments were repeated twice.

### Ethics statement

Ethics approval is not required from a committee for work with fruit flies, however every effort was made to minimize animal suffering.

### Statistical analysis

The logarithms of expression levels were calculated and the difference between groups was assessed by two-tailed independent-samples t-test (SPSS, Inc., Chicago, IL).

## Supporting Information

S1 Fig Sequences for constructs p37δ, N-Dp110 and Dp37, cloned into the pUASTattB-vector for expression in *Drosophila Melanogaster*. PCR primer sequences are underlined, start and stop-codons are indicated by red lettering and sequence corresponding intron 5 of human *PIK3CD* is capitalized. The stop-codon in N-Dp110 was introduced in the PCR.
(PDF)
