# Supplementary material for: The Unique Non-Catalytic C-Terminus of P37delta-PI3K Adds Proliferative Properties In Vitro and In Vivo
Source: PLoS One. 2015 May 29;10(5):e0127497. doi: 10.1371/journal.pone.0127497 (PMC4449119; doi:10.1371/journal.pone.0127497)
Supplement: S1 Fig — PCR primer sequences are underlined, start and stop-codons are indicated by red lettering and sequence corresponding intron 5 of human PIK3CD is capitalized. The stop-codon in N-Dp110 was introduced in the PCR. (PDF) [file pone.0127497.s001.pdf]

p37delta: *PIK3CD_v2*$^{p37\delta}$ Human

gagggggctttgctggtctttcttggactattccagagaggacaactgtcatctgggaagtaacaacgcaggATGccccctggggtggactgc
cccatggaattctggaccaaggaggagaatcagagcgttgtggttgacttcctgctgcccacaggggtctacctgaacttccctgtgtcccgc
aatgccaacctcagcaccatcaagcagctgctgtggcaccgcgcccagtatgagccgctcttccacatgctcagtggccccgaggcctatgtg
ttcacctgcatcaaccagacagcggagcagcaagagctggaggacgagcaacggcgtctgtgtgacgtgcagcccttcctgcccgtcctgcgc
ctggtggcccgtgagggcgaccgcgtgaagaagctcatcaactcacagatcagcctcctcatcggcaaaggcctccacgagtttgactccttg
tgcgacccagaagtgaacgactttcgcgccaagatgtgccaattctgcgaggaggcggccgcccgccggcagcagctgggctgggaggcctgg
ctgcagtacagtttcccccctgcagctggagccctcggctcaaacctggggggcctggtaccctgcggctcccgaaccgggcccttctggtcaac
gttaagtttgagggcagcgagGTGAGCCCATGCGTGGCCTGCGGCATCCAGGCTGCTCTGTCCATGGGGAGCACTTCCTCTGTGAAACTCCTC
AGTCATCCGCAAGCCCCCCTCCCCCAGTGGCATCAGATGGTGTTTGCCAGGTGTCTGTGCATGTGTGGGGCTCAACTGAATGTCCCCCCAGga
gagcttcaccttccaggtgtccaccaaggacgtgccgctggcgctgatggcctgtgccctgcggaagaaggccacagtgttccggcagccgct
ggtggagcagccggaagactacacgctgcaggtgaacggcaggcaTGAgtacctgtatg

N-Dp110: N-terminal *PiK92E* Drosophila

atccgaggcaccagatccaaaatccgaacttgaagatcaaatagtcATGaacatgatggacaaccgggcgttggcctacgtggcccaccagcc
caagtatgagacaccgccggaagaagcggagccgccctgcatgcgcttttcggttaacctgtggaaaaacgagatgctgaactgggtggacct
aatctgcctgttgcccaatggattcctgctggagctcagggtcaatccggccaacaccatccaggtaatcaaggtggagatggtcaaccaggc
caaacagatgccactgggctatgtgatcaaagaggcctgcgagtaccaggtgtacggcatctcgaccttcaacatcgaaccttacaccgacga
aacgaagcgactcagtgaggtccagccgtacttcggcatcctcagcctcggcgagcgcaccgacacaacgagctttagcagcgactacgagct
gaccaagatggttaacggaatgatcggcaccaccttcgatcataatcgaacgcacggctcgcccgagattgacgacttccggctgtatatgac
ccaaacttgcgacaacatcgaactggagcgctccgcctacacctggcagcagagactgctctacgagcatcctctgcgactggcgaactcgac
taaaatgcccgagctgatacgggagcggcatccgaccagaacctttctcatcgtcgtaaagaacgagaacgacTAGa

Dp37: N-terminal *PiK92E* Drosophila + C-terminal *PIK3CD_v2*$^{p37\delta}$ Human

atccgaggcaccagatccaaaatccgaacttgaagatcaaatagtcATGaacatgatggacaaccgggcgttggcctacgtggcccaccagcc
caagtatgagacaccgccggaagaagcggagccgccctgcatgcgcttttcggttaacctgtggaaaaacgagatgctgaactgggtggacct
aatctgcctgttgcccaatggattcctgctggagctcagggtcaatccggccaacaccatccaggtaatcaaggtggagatggtcaaccaggc
caaacagatgccactgggctatgtgatcaaagaggcctgcgagtaccaggtgtacggcatctcgaccttcaacatcgaaccttacaccgacga
aacgaagcgactcagtgaggtccagccgtacttcggcatcctcagcctcggcgagcgcaccgacacaacgagctttagcagcgactacgagct
gaccaagatggttaacggaatgatcggcaccaccttcgatcataatcgaacgcacggctcgcccgagattgacgacttccggctgtatatgac
ccaaacttgcgacaacatcgaactggagcgctccgcctacacctggcagcagagactgctctacgagcatcctctgcgactggcgaactcgac
taaaatgcccgagctgatacgggagcggcatccgaccagaacctttctcatcgtcgtaaagaacgagaacgaccagGTGAGCCCATGCGTGGC
CTGCGGCATCCAGGCTGCTCTGTCCATGGGGAGCACTTCCTCTGTGAAACTCCTCAGTCATCCGCAAGCCCCCCTCCCCCAGTGGCATCAGAT
GGTGTTTGCCAGGTGTCTGTGCATGTGTGGGGCTCAACTGAATGTCCCCCCAGgagagcttcaccttccaggtgtccaccaaggacgtgccgc
tggcgctgatggcctgtgccctgcggaagaaggccacagtgttccggcagccgctggtggagcagccggaagactacacgctgcaggtgaacg
gcaggcaTGAgtacctgtatgg

Figure S1. **Sequences for constructs p37delta, N-Dp110 and Dp37, cloned into the pUASTattB-vector for expression in *Drosophila Melanogaster*.** PCR primer sequences are underlined, start and stop-codons are indicated by red lettering and sequence corresponding intron 5 of human *PIK3CD* is capitalized. The stop-codon in N-Dp110 was introduced in the PCR.
